# Modification of Natural Clays with Magnetite to Provide Boosted Antimicrobial Properties and Chemopreventive Activity Against Melanoma

**DOI:** 10.3390/ma18204759

**Published:** 2025-10-17

**Authors:** Alicja Wójcik, Jakub Matusiak, Marta Trzaskowska, Aleksandra Maciejczyk, Paulina Kazimierczak, Katarzyna Suśniak, Krzysztof Palka, Izabela Korona-Glowniak, Wojciech Franus, Agata Przekora

**Affiliations:** 1 Department of Tissue Engineering and Regenerative Medicine, Medical University of Lublin, 20-093 Lublin, Poland; alicja.wojcik1@umlub.edu.pl (A.W.); marta.trzaskowska@umlub.edu.pl (M.T.); aleksandra.maciejczyk@umlub.pl (A.M.); paulina.kazimierczak@umlub.pl (P.K.); 2 Department of Construction Materials Engineering and Geoengineering, Lublin University of Technology, 20-618 Lublin, Poland; j.matusiak@pollub.pl (J.M.); w.franus@pollub.pl (W.F.); 3 Department of Pharmaceutical Microbiology, Medical University of Lublin, 20-093 Lublin, Poland; katarzyna.susniak@umlub.pl (K.S.); izabela.korona-glowniak@umlub.pl (I.K.-G.); 4 Department of Materials Engineering, Lublin University of Technology, 20-618 Lublin, Poland; k.palka@pollub.pl

**Keywords:** nanostructured materials, kaolinite, glauconite, montmorillonite, bentonite, cytotoxicity, wound healing

## Abstract

Historically, clays have been widely used for the treatment of wounds and to stop hemorrhaging. The aim of this study was to combine four natural clay minerals (kaolinite, glauconite, montmorillonite, and bentonite) with magnetite (Fe_3_O_4_) nanoparticles to produce Fe_3_O_4_–clay complexes with enhanced antimicrobial properties and chemopreventive activity against melanoma. The magnetite–clay complexes were synthesized by the chemical co-precipitation method and characterized using XRD, TEM, STEM-EDS, SEM, and SQUID magnetometer. Antimicrobial properties were determined by evaluation of MIC values. The most promising materials were also subjected to direct contact antibacterial test according to the OECD standard for porous materials. Cytotoxicity of the complexes towards melanoma cells and normal human skin fibroblasts was assessed by MTT assay. We performed XRD, which confirmed the formation of Fe_3_O_4_–clay complex materials. It was also proven that complexes exhibited superparamagnetic properties. Microbiological experiments clearly revealed that modification of natural clays with magnetite significantly boosted their antimicrobial properties. Fe_3_O_4_–montmorillonite and Fe_3_O_4_–bentonite showed the strongest antimicrobial activity. Moreover, the mentioned complexes had the ability to reduce the viability of melanoma cells by 35–40%, while exhibiting no cytotoxicity against the normal human fibroblast (BJ) cell line, which is an extremely desirable feature. Thus, it may be concluded that Fe_3_O_4_–montmorillonite and Fe_3_O_4_–bentonite complexes hold promise for use in the management of infected wounds and wounds after melanoma excision.

## 1. Introduction

The skin is the largest organ of the body and is responsible for a number of important functions; the main one is to provide a protective barrier against unfavorable environmental conditions [1]. A wound is the consequence of a break in the continuity of the skin covering. This leads to disruptions in the functionality of skin and—in extreme cases—can lead to life-threatening conditions [2]. Chronic wounds are a significant medical and socioeconomic burden, affecting over 6.5 million individuals in the United States alone. This results in billions of dollars in healthcare costs annually, posing a significant challenge to the public health system [3]. Unlike acute wounds, which typically follow a predictable healing trajectory, chronic wounds are characterized by a prolonged inflammatory phase that impairs tissue regeneration. The treatment of such wounds remains a significant clinical challenge due to their complex etiology, prolonged healing time, and high risk of complications. These wounds can be caused by trauma or burns but often are associated with underlying comorbidities such as diabetes, venous stasis, bedsores, or wounds resulting from oncological procedures. Chronic wounds are characterized by impaired inflammatory response, disrupted tissue regeneration, and persistent infections. Effective management of chronic wounds requires a multifactorial approach combining infection control, modulation of inflammation, and stimulation of tissue repair [4,5]. When searching for new, innovative methods of treating chronic wounds, it is important to remember the growing problem of antibiotic resistance. For this reason, searching for new agents with antimicrobial properties is absolutely justified and should be taken under the highest consideration [6]. Recently, there has been great interest in searching for new, non-antibiotic methods of combating microorganisms that cause infections. This is related to the emerging global problem of multidrug resistance among bacteria [7]. Metals such as silver [8], copper [9], aluminum [10], zinc [11,12], and iron [13] deserve special attention in the context of antimicrobial activity. Therefore, the development of innovative, multifunctional materials enriched with metal nanoparticles or their oxides which are capable of accelerating healing and preventing infection has become a priority in regenerative medicine.

Iron oxide nanoparticles, particularly magnetite (Fe_3_O_4_), exhibit superparamagnetic behavior and are well-known for their strong antimicrobial effects mediated through reactive oxygen species (ROS), which damage bacterial cell walls, proteins, and DNA [14]. Studies have shown that Fe_3_O_4_ is effective against a wide range of microorganisms, including antibiotic-resistant strains of *Escherichia coli* and *Staphylococcus aureus* [15]. Given the rising prevalence of antibiotic resistance, the local administration of antiseptics represents a promising alternative for the treatment of infected wounds, as they are more effective during the initial phase of wound healing and do not contribute to the development of bacterial resistance, which is an excellent strategy for protecting wounds from excessive growth of microorganisms.

In turn, clay is a naturally occurring material rich in minerals, typically containing components such as carbonates, silicates, hydrated iron, and aluminum oxides, along with various organic and inorganic substances. Common clay types applied in biomedical contexts include kaolinite, halloysite, montmorillonite, beidellite, talc, sepiolite, and palygorskite [16]. Historically and in traditional medicine, natural clays have been extensively utilized for wound management and bleeding control [17,18]. Their antimicrobial properties are attributed to the presence of metal ions and complexes that can attract toxins and absorb pathogens, thereby promoting the healing of infected wounds [16,19]. Additionally, clays can influence environmental parameters in the wound bed such as pH, redox potential, and osmotic pressure, which enhance their bactericidal action while maintaining the viability of surrounding eukaryotic cells [16]. As a result, clay-based components are frequently found in wound dressing formulations. Some studies highlight the development of hemostatic materials by integrating clays such as montmorillonite, kaolinite, and halloysite with polymers such as chitosan, polyvinyl pyrrolidone (PVP), polyethylene terephthalate (PET), and cotton [20].

Beyond their antimicrobial functions, clays and iron oxide nanoparticles are gaining interest for their anticancer properties. Fe_3_O_4_ has been shown to exert selective toxicity against cancer cells via ROS-mediated mechanisms. Thus, it may be assumed that after its incorporation into biomaterial, Fe_3_O_4_ may contribute to local tumor suppression following surgical resection. For patients with a history of skin cancers, such as melanoma, application of Fe_3_O_4_-loaded wound dressings may potentially offer an additional benefit by reducing the risk of local recurrence at the wound site after cancer excision. It should be noted that surgical excision with extended margins is the primary treatment of localized skin cancers (including melanoma); nevertheless, there is a risk of remaining residual cancer cells, causing a high local recurrence risk in skin cancer therapy [21,22,23]. Therefore, it was hypothesized that the integration of Fe_3_O_4_ nanoparticles with natural clay minerals may lead to the formation of a multifunctional complex with enhanced antibacterial properties and potential chemopreventive efficacy. Additionally, resultant complexes will be characterized by superparamagnetic properties, enabling their potential activation via magnetic field, which is known to promote microcirculation and wound healing [24,25].

The aim of this study was to synthesize and characterize Fe_3_O_4_-loaded clays, which can be potentially used as a component of dressing materials designed for the management of chronic and infected wounds. Moreover, it was assumed that the selective toxicity of Fe_3_O_4_ nanoparticles towards cancer cells may provide an additional benefit of magnetite–clay complexes in the treatment of post-oncological skin wounds, where the complex could significantly reduce viability of remaining tumor cells after skin cancer resection, providing a chemopreventive effect. The results presented here provide a rationale for further development of multifunctional and magneto-responsive wound dressings as alternatives to conventional therapies for wounds.

## 2. Materials and Methods

### 2.1. Synthesis of Magnetite–Clay Complexes

**Figure 1:**
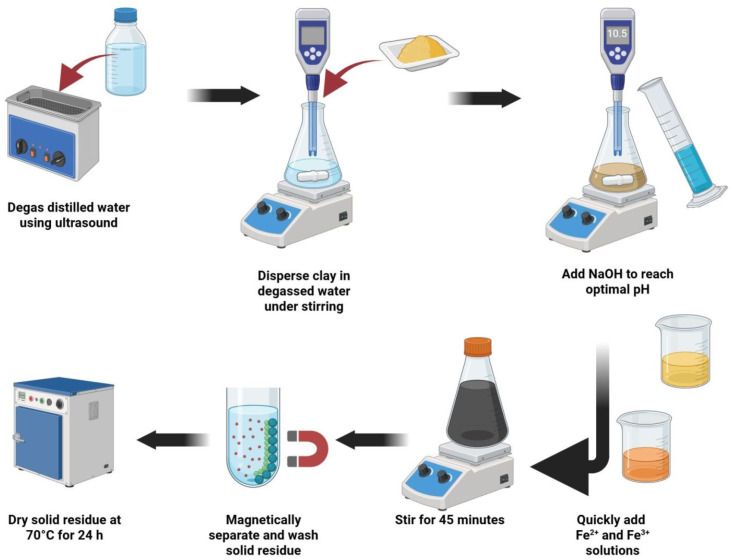
Schematic representation of the synthesis of magnetite–clay composites via co-precipitation. Created in BioRender. Matusiak, J. (2025) https://BioRender.com/pimxgjs.

Magnetite–clay composites (Fe_3_O_4_–clay) were synthesized via co-precipitation under aqueous conditions at room temperature in an air atmosphere. A graphical representation of the synthesis is shown in Figure 1. The distilled water used in all steps was degassed by ultrasound treatment for 10 min prior to synthesis. A homogeneous aqueous suspension of the selected clay (2 g of kaolinite, glauconite, montmorillonite, or bentonite) was placed in a capped laboratory flask on a magnetic stirrer. Glauconite and montmorillonite were acquired from natural sources and were collected in Poland. Glauconite was sourced from glauconitic sands mined in the vicinity of Nowodwór (eastern Poland) [26], whereas montmorillonite was obtained from clinoptilolite–montmorillonite mudstones located near Dylągówka (southeastern Poland) [27]. The obtained minerals were thoroughly washed with ultrapure water, dried, and powdered before use. Kaolinite and bentonite were purchased from Sigma-Aldrich (Warsaw, Poland). Prior to use, all clays were washed repeatedly with ultrapure water until the conductivity of the supernatant was below 100 μS/cm. A specific volume (60–70 mL) of degassed aqueous NaOH solution (1 M) was then added to adjust the pH to 10–10.5. The resulting solid-to-liquid ratio at this step was therefore in the range of 1:41.25–1:42.5. Subsequently, iron(II) and iron(III) chloride salts, dissolved in degassed water at a 2:3 molar ratio, were rapidly added to the alkaline clay suspension.

The resulting black colloid was stirred for 45 min, after which the solid residue was magnetically separated and washed with water to remove residual synthesis byproducts. The final powder was dried at 70 °C for 24 h.

The reagents used during the synthesis (NaOH, FeCl_2_*4H_2_O, and FeCl_3_*6H_2_O) were of an analytical grade (>98% purity) and were purchased from Chempur (Piekary Śląskie, Poland).

### 2.2. Characterization of Magnetic Complexes

The Fe_3_O_4_ particle size and the elemental composition were examined by scanning transmission electron microscopy (STEM) with energy-dispersive spectroscopy (EDS). The structural and morphological properties of the Fe_3_O_4_-clay complexes were characterized using X-ray diffraction (XRD) and magnetization measurements were performed with a SQUID magnetometer. The morphology of native and modified clays was also studied using scanning electron microscopy (SEM).

#### 2.2.1. TEM and STEM-EDS Analysis

High-resolution transmission electron microscopy (TEM) and scanning transmission electron microscopy with energy-dispersive X-ray spectroscopy (STEM–EDS) were performed using a Titan G2 60–300 kV microscope (FEI Company, Hillsboro, OR, USA). The samples were prepared by pipetting a dilute ethanol-based slurry onto 300-mesh copper grids coated with lacey formvar and stabilized with carbon, followed by solvent evaporation on filter paper. Elemental mapping was carried out in STEM-EDS mode and the resulting maps were visualized as color-coded matrices, with the signal intensity corresponding to the elemental distribution.

#### 2.2.2. SEM Imaging

The morphology of native and modified clays were studied by scanning electron microscopy (SEM) using Nova NanoSEM 450 (FEI, Eindhoven, The Netherlands). The samples were analyzed under low-vacuum mode without any prior preparations. The images were acquired by low vacuum secondary electron detector at a working distance of 10.4 mm and 4.5 spot size. The magnification used in all the shown SEM images was 2000×.

#### 2.2.3. X-Ray Diffraction Analysis

X-ray diffraction (XRD) analysis was performed using a Panalytical X’pert PRO MPD diffractometer (Malvern Panalytical, Brighton, UK) equipped with PIXcel 3D detector and Cu Kα radiation source operating at 40 kV and 30 mA. Diffraction patterns were collected over a 2θ range of 5–65° with a step size of 0.02°. Powdered samples were lightly pressed onto standard sample holders for measurement. The results were analyzed using X’Pert Highscore software 4.8 (Malvern Panalytical, Almelo, The Netherlands) with the PDF-2 release 2010 database, formalized by JCPDS-ICDD.

#### 2.2.4. Magnetization Measurements

Magnetic measurements were carried out at 300 K using an MPMS3 SQUID magnetometer (Quantum Design, San Diego, CA, USA). Magnetization curves (M-H) were recorded by varying the applied magnetic field in the range of −70,000 to +70,000 Oe. Samples were measured in powder form using standard non-magnetic sample holders. The saturation magnetization (Ms) was estimated by averaging the magnetization values in the high-field region where the curve plateaued, using the last 10 data points in the Excel dataset.

### 2.3. Antimicrobial Activity Tests

#### 2.3.1. MIC Value Determination

The antibacterial and antifungal activity of clays and magnetite–clay complexes was assessed using their suspensions in a growth medium. The micro-dilution broth method was applied according to the guidelines of the European Committee on Antimicrobial Susceptibility Testing (EUCAST) (www.eucast.org; accessed on 1 March 2021). Mueller-Hinton (M-H, BioMaxima, Lublin, Poland) broth was used as the growth medium for bacteria, and M-H broth with 2% glucose was used for fungi. The minimum inhibitory concentration (MIC) was determined for the Gram-negative bacteria *Escherichia coli* ATCC 25922 and *Pseudomonas aeruginosa* ATCC 9027, the Gram-positive bacteria *Staphylococcus aureus* ATCC 25923, and the fungus *Candida albicans* ATCC 10231 (ATCC-LGC Standards, Teddington, UK). Clay dilutions ranging from 20 to 0.156 mg/mL were prepared by serial two-fold dilutions in the broth medium. Then, 100 µL of the appropriate dilution was transferred to the wells of a 96-well plate. Dilutions of fresh microbial cultures at 0.5 McFarland density in sterile 0.85% NaCl were prepared and added to the wells, resulting in a final density of 5 × 10^5^ CFU/mL for bacteria and 5 × 10^4^ CFU/mL for fungus. The plates were incubated for 24 h at 35 °C. A negative control (containing clay suspensions without inoculum) and a positive control (containing inoculum without clay suspensions) were also included on each plate. The MIC, defined as the lowest concentration of the suspension that prevented visible growth of the microorganism, was determined using a spectrophotometer at a wavelength of 600 nm. The minimum bactericidal concentration (MBC) or minimum fungicidal concentration (MFC) was evaluated by culturing 5 µL of microbial suspension (on agar plates) from each well in which complete inhibition of growth by clay was observed. The plates were then incubated for 24 h at 35 °C. MBC/MFC corresponds to the lowest concentration of the clay suspension at which no growth of microorganisms was observed. The bacteriostatic/fungistatic or bactericidal/fungicidal nature of antimicrobial activity was determined using the MBC/MIC ratio.

#### 2.3.2. Direct Contact Antibacterial Test

The antibacterial activity of the clays was assessed by quantifying bacterial viability following direct contact with the material. The experimental procedure was performed in accordance with OECD guideline No. 202 (JT03360420) [28] for porous materials. Test samples (50 mg each) were evaluated in triplicate, while the control material (lacking antibacterial properties) was tested in six replicates—three for determining the colony-forming units (CFU) immediately after bacterial inoculation and three after a 24 h incubation period. Commercial hydroxyapatite granulate sintered at high temperature (HA BIOCER, Chema Elektromet, Rzeszow, Poland) was used as a control material. First, a 1:50 dilution of M-H broth in water (marked 1/50 M-H broth) was prepared. This M-H broth dilution was selected based on preliminary experiments, as the 1:500 dilution recommended by the OECD did not guarantee sufficient bacterial survival on the control samples after 24 h. Then, the bacterial inoculum was prepared by diluting a 0.5 McFarland standard bacterial suspension 1:250 in 1/50 M-H broth. The final inoculum concentration was approximately 6 × 10^5^ CFU/mL. Each sample was pre-soaked with 25 µL of 1/50 MH broth, followed by the inoculation of 25 µL of the bacterial suspension. The samples were incubated at 37 °C for 24 h. Half of the control samples were analyzed immediately after inoculation, while the remaining were processed post-incubation. After the 24 h incubation, Eugon LT 100 broth neutralizer (BTL, Warsaw, Poland) was added to the samples, which were then vigorously agitated to release bacteria settled on the material. The number of viable bacteria was determined using serial dilutions and the pour plate method. Agar plates were incubated for 24 h at 37 °C, and bacterial colonies were enumerated using a Scan 300 automated colony counter. The antibacterial effectiveness of the materials was expressed as both a percentage reduction and a logarithmic reduction in bacterial count. The following formulas were used for calculations:

*Log reduction* = log (A) − log (B)

$$Percent\;reduction=\frac{\left(A-B\right)\times 100}{A}$$

where

A is the number of viable bacteria from the control sample after 24 h of incubation.

B is the number of viable bacteria from the test sample after 24 h of incubation.

### 2.4. Cell Culture Experiments

#### 2.4.1. Cells

Human normal skin fibroblasts (BJ cell line, CRL-2522™) and human melanoma cells (A375 cell line, CRL-1619™) obtained from the American Type Culture Collection (ATCC-LGC Standards, Teddington, UK) were utilized in the cell culture experiments. The BJ cells were cultured in Eagle’s Minimum Essential Medium (30-2003, ATCC-LGC Standards, Teddington, UK) supplemented with 10% fetal bovine serum (FBS; Pan-Biotech GmbH, Aidenbach, Bavaria, Germany), 100 U/mL penicillin, and 0.1 mg/mL streptomycin (Sigma-Aldrich Chemicals, Warsaw, Poland). The A375 cells were cultured in Dulbecco’s Modified Eagle’s Medium (30-2002, ATCC-LGC Standards, Teddington, UK) supplemented with 10% fetal bovine serum (FBS; Pan-Biotech GmbH, Aidenbach, Bavaria, Germany), 100 U/mL penicillin, and 0.1 mg/mL streptomycin (Sigma-Aldrich Chemicals, Warsaw, Poland). Cells were maintained at 37 °C in a humidified atmosphere containing 5% CO_2_.

#### 2.4.2. Cytotoxicity Assay

Cytotoxicity assessment was performed in accordance with the standardized protocol outlined in ISO 10993-5 [29]. Cells were seeded into 96-well plates at a density of 2 × 10^4^ cells per well in 100 μL of culture medium and incubated for 24 h. Subsequently, the culture medium was replaced with sample extracts prepared according to ISO 10993-12 [30] by incubating the materials in complete culture medium at a ratio of 100 mg of material per 1 mL of medium at 37 °C for 24 h. Polypropylene extract served as the negative cytotoxicity control. Cells were exposed to the material extracts for 24 h, after which cell viability was assessed using the MTT assay (Sigma-Aldrich Chemicals, Poland). Results were expressed as a percentage relative to the absorbance value of the negative control.

### 2.5. Statistical Analysis

The data were presented as mean values ± standard deviation (SD) based on at least three independent experiments (n = 3). Statistical analysis was performed using one-way ANOVA followed by Dunnett’s post hoc test, with significance set at *p* < 0.05 (GraphPad Prism 8.0.0; GraphPad Software Inc., San Diego, CA, USA).

## 3. Results and Discussion

### 3.1. Characterization of Magnetite–Clay Complexes

**Figure 2:**
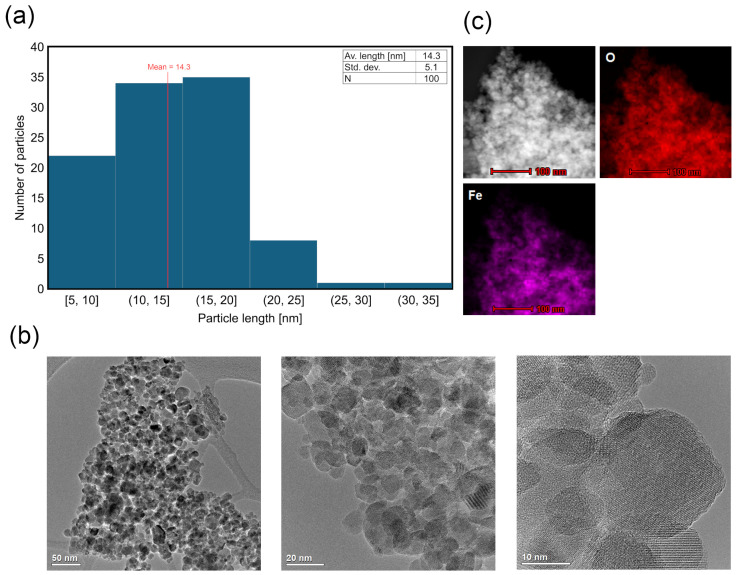
TEM micrographs of magnetite nanoparticles: (**a**) overview of particle length distribution; (**b**) high-magnification image revealing morphology of the nanoparticles; (**c**) STEM-EDS mapping showing the elemental composition.

To establish the optimal synthesis conditions of the magnetite–clay complexes, the initial step was the synthesis of magnetite alone. Among the different methods used to synthesize iron oxide (Fe_x_O_y_) nanoparticles, co-precipitation is one of the most popular methods due to its simplicity and scalability. However, the process is sensitive to parameters such as pH, Fe(II)/(III) ratio, mixing rate, and atmosphere; thus, establishing appropriate conditions is essential. In this study, the goal was to synthesize magnetite nanoparticles in accordance with sustainable development principles, while maintaining high efficiency. Thus, the synthesis did not require additional heating and was carried out in an air atmosphere. As shown in Figure 2a, the resulting nanoparticles exhibited an average diameter of 14.3 nm, with the largest population having diameters between 10 and 20 nm. HR-TEM images showed that the particles exhibited a near-spherical morphology with moderate size uniformity and slight agglomeration, which is typical of magnetic interactions (Figure 2b). The STEM-EDS elemental mapping (Figure 2c) confirmed the presence of Fe and O in regions corresponding to the nanoparticulate domains, which is consistent with the formation of magnetite. No signals indicative of other phases or contaminants were detected.

**Figure 3:**
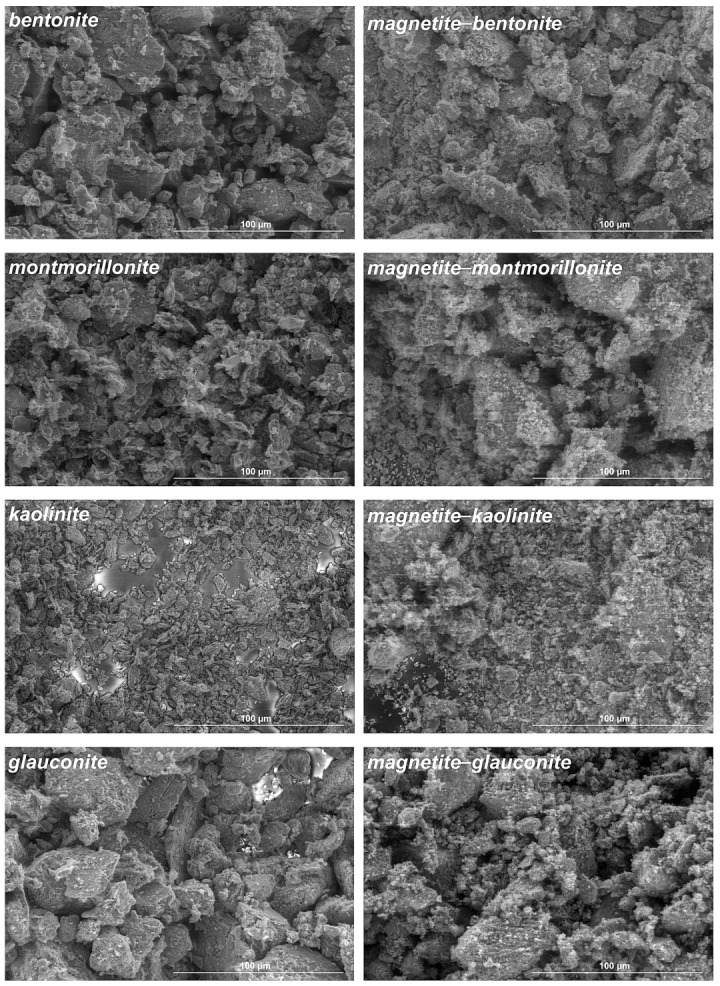
Comparison of the surface morphology of native clays and magnetite–clay complexes by SEM. Magnification 2000×.

Evaluation of the surface morphology changes before and after the functionalization of native clays was carried out using SEM (Figure 3). The structures of bentonite and montmorillonite were observed to be similar, which is consistent with the mineralogical composition of bentonite being predominantly montmorillonite. In all the studied materials, magnetic modification led to the formation of a magnetite–clay complexes composed of clay particles decorated with magnetite particles. The extent and distribution of magnetite coverage varied slightly between clay types. This was possibly because of differences in surface charge, morphology, or layer structure influencing the nucleation of iron oxide particles. SEM analysis showed that the morphologies of bentonite, montmorillonite, kaolinite, and glauconite were preserved after functionalization. No fragmentation or macroscopic collapse of the clay particles was observed. This could indicate that the co-precipitation method maintained overall particle integrity. We observed that aggregation was more pronounced in the complexes, which is consistent with surface coverage by magnetite nanoparticles. The magnetite was visible as nanoscale deposits decorating the platelet surfaces, with differences in coverage reflecting the mineralogical variability of the native clays. It should be noted, however, that SEM provides information at the micron scale; thus, while particle shapes appear intact, disruptions in long-range basal stacking order—as indicated by the disappearance of the (001) reflection in the XRD for the bentonite composite—are not detectable in SEM images.

**Figure 4:**
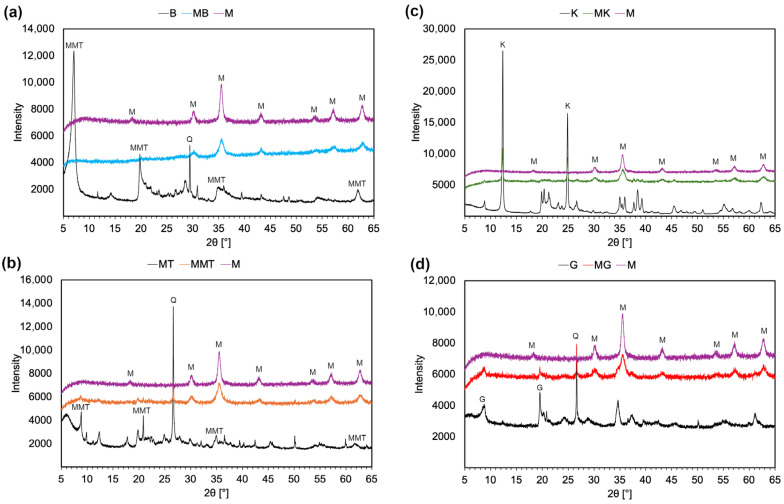
X-ray diffraction (XRD) patterns of native clays, magnetically modified clays, and reference magnetite (M): (**a**) bentonite (B) and magnetically modified bentonite (MB); (**b**) montmorillonite (MT) and magnetically modified montmorillonite (MMT); (**c**) kaolinite (K) and magnetically modified kaolinite (MK); (**d**) glauconite (G) and magnetically modified glauconite (MG); Q—quartz.

X-ray diffraction (XRD) was used to study the structure of the synthesized materials and the efficiency of magnetic modification. Figure 4 shows diffractograms of native and modified clay materials along with magnetite (M) for comparison. Under optimized conditions, XRD confirmed the formation of crystalline, single-phase magnetite. The observed peaks at about 2θ° of 18.32, 30.18, 35.59, 43.18, 53.54, 57.13, and 62.72 are aligned with magnetite, as reported in JCPDS #19-0629 [31]. Additional peaks corresponding to maghemite or hematite were not observed, confirming the high phase purity of magnetite within the detection limits of XRD [32]. Bentonite is a claystone composed of different minerals, the most abundant being montmorillonite [33]. Figure 4a confirms that the native bentonite used in this study contained montmorillonite, quartz, and other clay components. It was previously observed that those are usually cristobalite, calcite, illite, and feldspar [34]. In magnetically modified bentonite, both Fe_3_O_4_ and bentonite components (montmorillonite, quartz, and other) were observed. However, the strongest reflection of montmorillonite at 2θ between 6 and 8° (001) was absent in the magnetically modified bentonite. This can be attributed to the disruption of the interlayer structure, partial structural collapse, or shielding effects caused by the surface-deposited layer of magnetite particles. In the bentonite complex, the disappearance of the (001) reflection is unlikely to have resulted from interlayer ion exchange, since the synthesis was carried out under conditions designed to avoid that problem. A more plausible explanation is that the high pH and ionic strength, combined with rapid magnetite precipitation on platelet surfaces and edges, may have disturbed the regular parallel stacking of layers and thereby weakened or eliminated the basal reflection. In addition, a magnetite overlayer could have contributed by masking weak low-angle signals. Because pristine bentonite already shows a broader and less well-defined (001) than purified montmorillonite, these factors together may explain why the basal reflection fell below detectability in the bentonite composite. For purified montmorillonite, a more uniform composition and sharper basal reflection is expected, which likely allowed it to retain the stacking order for the (001) after functionalization. As stated above, montmorillonite is a mineral of the smectite group that is a part of the bentonite structure. Consequently, in the montmorillonite sample, as for bentonite, some inevitable impurities of other clay minerals and quartz were observed (Figure 4b). After magnetic modification, the montmorillonite structure was retained. The unchanged position of the (001) peak indicated that magnetic particles were co-precipitated on the surface rather than intercalated into the layered structure of the mineral. The analysis of the kaolinite sample revealed (Figure 4c) the typical structure of this mineral, with the two strongest reflections observed around 2θ ≈ 12.37° and 24.87°, corresponding to the (001) and (002) planes, respectively [35]. In the kaolinite–magnetite complexes, the crystalline structure was retained, which confirmed that the basal layer was not destroyed and was decorated with magnetite. The analysis of glauconite confirmed the presence of a basal reflection (001) at about 2θ ≈ 8° and a (002) plane at about 2θ ≈ 17.7° [36]. Some impurities of quartz and other clay-minerals were also observed (Figure 4d). The XRD data confirmed that the magnetic modification was also successful.

**Figure 5:**
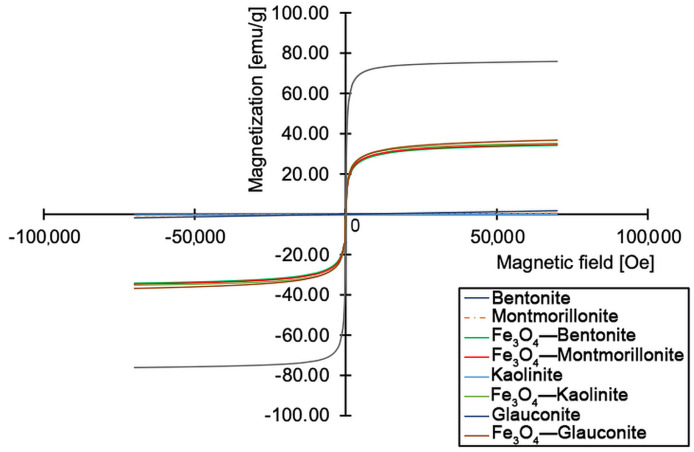
The M-H curves of native and modified clays.

Magnetic parameters for native magnetite and magnetite–clay complexes were studied using SQUID type magnetometer (Figure 5). The M-H curve of magnetite was characterized by a narrow hysteresis loop with a sigmoidal shape typical for superparamagnetic behavior. These kinds of materials are characterized by very low or zero remanence or coercivity [37]. This property is very important for biomedicine because nanoparticles used for medical applications must not be characterized by permanent magnetic properties due to the negative influence of this on living organisms. In the case of superparamagnetic materials, they are susceptible to magnetic fields but return to a normal state when the influence of the field is diminished. This allows for the development of personalized devices and therapies without causing negative long-term effects related to magnetic fields. The value of M_s_ obtained (M_s_ = 75.6 emu/g) was high and comparable to data from the literature [38], which indicates a well-crystallized magnetic phase, consistent with pure nano-magnetite. The non-magnetic clays alone exhibited near-zero magnetization, as seen based on their flat baseline curves. All magnetite–clay complexes showed lower magnetization in comparison to native magnetite, reflecting the dilution effect caused by the non-magnetic clay matrix. Nonetheless, these materials were characterized by superparamagnetic behavior due to the presence of magnetite nanoparticles on the clay surface; therefore, they could be further considered for biomedical applications.

### 3.2. Antimicrobial Activity Assessment

In order to assess the antimicrobial properties of clays and clay–magnetite complexes, their suspensions were tested in the concentration range of 0.156–20 mg/mL using the microdilution method. Pure clays showed MIC values ranging from 2.5 to 20 mg/mL for the tested bacterial strains and *C. albicans*. Although the traditional use of clays in wound management as healing-promoting agents is well documented [17], our results indicate that not all clays exhibit antimicrobial activity, which is consistent with previous studies [39,40,41]. Among tested clays, bentonite was proven to be the clay with the highest bacteriostatic activity. Thus, it may be potentially used as a promising component of dressing materials with antimicrobial activity. The MIC value obtained for bentonite was in the range of 10–20 mg/mL for Gram-negative bacteria and 2.5 mg/mL for Gram-positive bacteria (Table 1).

In the case of the other clays, no activity against Gram-negative bacteria was noted in the tested concentration range. For Gram-positive bacteria, the MIC was in the range of 2.5–10 mg/mL. On the other hand, montmorillonite was the clay with the most fungicidal activity against *C. candida* (MBC/MIC = 1). The bacteria most sensitive to the bacteriostatic activity of pure clays was Gram-positive *S. aureus*, with an MIC in the range of 2.5–10 mg/mL. This is especially valuable information because *S. aureus* is known to evolve resistance mechanisms against all available antibiotic classes, with de novo mutations contributing to this process, so the search for antibacterial substances that show activity against these bacteria is particularly important and could be beneficial in wound treatment [42].

Modification of clays with magnetite significantly improved their antimicrobial activity with an MIC in the range of 0.156–20 mg/mL for Gram-negative and positive bacteria and 1.25–5 mg/mL for *C. albicans*. The highest bacteriostatic activity was demonstrated by the Fe_3_O_4_–montmorillonite complex, with an MIC of 0.156 mg/mL for all tested bacterial strains. It is not surprising that the enrichment of clay with magnetite leads to an increase in its antimicrobial activity. Morrison et al. [43] investigated the bactericidal properties of blue clay and demonstrated that, alongside aluminum, iron is the main bactericidal element in Oregon blue clay. Similarly, Azmi et al. [44] demonstrated the presence of magnetite in Carey clay, which exhibited activity against *S. aureus* and showed a significant reduction in bacterial viability.

The good antimicrobial properties of the Fe_3_O_4_–montmorillonite complex which have been presented here are supported by the findings of other researchers. Jee et al. [45] showed that the Fe_3_O_4_–montmorillonite–tannic acid complex is a potential excellent antimicrobial carrier against *S. aureus*. It should be noted that the assessment of the antimicrobial activity of iron oxide nanoparticles has been widely described in the literature. Qasim et al. [46] demonstrated that iron oxide nanoparticles may exhibit antibacterial activity against *Pseudomonas aeruginosa* with proven resistance to multiple antibiotics. Similarly, Abdelghany et al. [47] demonstrated strong antibacterial activity of iron oxide nanoparticles against Gram-negative bacteria. In our study, the MIC of the Fe_3_O_4_–montmorillonite complex against Gram-negative bacteria was comparable to that of methylglyoxal—an active agent from Manuka honey [48] which is widely used in the treatment of chronic wounds. It can therefore be assumed that the aforementioned clay may have comparable antibacterial activity to Manuka honey in treating infected wounds, which is a very promising feature.

Importantly, in the case of *C. albicans*, the Fe_3_O_4_–kaolinite (MFC = 5 mg/mL), Fe_3_O_4_–glauconite (MCF = 5 mg/mL), and Fe_3_O_4_–montmorillonite (MCF = 1.25 mg/mL) complexes demonstrated fungicidal activity, as indicated by the MBC/MIC index ≤ 4. The obtained results clearly indicate that enrichment of clay with Fe_3_O_4_ nanoparticles boosted the antimicrobial properties of the clays used. This is in line with the results obtained by Long et al., who developed a bifunctional hybrid fiber membrane composed of ZnO–Fe_2_O_3_/kaolinite nanoclay/poly-(3-caprolactone)-gelatin with the ability to control bleeding, prevent bacterial colonization, reduce excessive inflammation, and facilitate wound healing in bacteria-infected mouse models [49].

Two clays and their modifications with the highest antimicrobial activity in the MIC assay were selected for a direct contact antibacterial test conducted according to the OECD standard. The activity of the clays montmorillonite, Fe_3_O_4_–montmorillonite, bentonite, and Fe_3_O_4_–bentonite was tested against Gram-positive and Gram-negative strains of bacteria related to wound infections: *S. aureus* and *E. coli*. Activity was assessed by determining the reduction in the number of bacteria on the tested materials compared to a material without any antibacterial activity. Hydroxyapatite granulate sintered at high temperatures, which is also a highly biocompatible ceramic, was selected as the control material. Montmorillonite and Fe_3_O_4_–montmorillonite clays demonstrated the highest bactericidal activity against both tested strains. The bacterial inoculum after exposure to these materials was reduced by 99.99% (log reduction of 4.07 for *S. aureus* and 6.8 for *E. coli*), which was equivalent to the elimination of all viable bacteria (Table 2). For bentonite and Fe_3_O_4_–bentonite clays, a 20.31% and 96.19% reduction in viable *E. coli* bacterial cells was observed, respectively. This indicated that modification with Fe_3_O_4_ significantly improved the bacteriostatic activity of bentonite against Gram-negative bacteria. However, no bacteriostatic effects of bentonite and Fe_3_O_4_–bentonite were observed against Gram-positive *S. aureus*. Interestingly, in a direct contact test, unmodified montmorillonite possessed high antibacterial activity. It is known that natural clays contain elements like aluminum, magnesium, iron, and their oxides. Thus, it was assumed that the iron oxide and titanium dioxide present in montmorillonite could be related to its antibacterial properties [50,51].

Maryan et al. also studied the antibacterial activity of montmorillonite and its modification (applying a quaternary ammonium salt) using the MIC method and in a direct contact test according to the AATCC 100-2004 standard [52,53]. The researchers observed antibacterial activity of the clay itself, especially in the direct contact test. The percentage reductions after exposure to montmorillonite for *S. aureus* and *E. coli* were 83% and 80.5%, respectively. However, the lowest inhibitory concentration was above the tested concentration range (>16,384 μg/mL). Due to the modification, the MIC values decreased to 4096 μg/mL and 5051 μg/mL for *E. coli* and *S. aureus*, respectively. Moreover, other authors modified mormorillonite with copper and silver ions. Hu et al. showed that montmorillonite had no antibacterial activity against *Aeromonas hydrophila*, while the MIC and MBC values for clay modified with Cu^2+^ ions were 150 and 600 mg/L, respectively [54]. Montmorillonite enriched with silver ions also demonstrated good antibacterial activity against *E. coli* [55].

The difference in the effectiveness of montmorillonite and bentonite clays, and their modifications, in the two microbiological experiments may be due to the different experimental conditions. The MIC test was conducted in a complete, liquid, bacterial culture medium in which the clays were suspended. In the direct contact test, bacteria were inoculated onto the surface of the clay only, which was moistened with a medium diluted 50× with distilled water. Therefore, different mechanisms could be responsible for the clays’ antimicrobial activity in the two mentioned microbiological experiments (i.e., MIC determination and direct contact test). Abdullayev et al. also observed differences in the antibacterial activity of clays depending on the applied test conditions, i.e., the solution in which the clay was suspended. The researchers pointed out that the key factor in this context was the pH of the solution, the lowering of which promoted the release of toxic metals [56]. Furthermore, other researchers showed that, depending on the experimental conditions, either Gram-negative [57] or Gram-positive [58,59] bacteria were more sensitive to the activity of clays. Referring to the results presented in this article, in the liquid medium (MIC experiment), factors released into the solution (e.g., metal ions) likely played a greater role in antibacterial activity [60]. However, in direct contact tests with the materials, charges on the clays’ surfaces or the clays’ ability to adhere to bacterial surfaces, which may reduce cell permeability, could have been more important [52]. In the context of physical interactions, clays exhibited strong adhesion to bacterial surfaces, which likely limited nutrient absorption and the removal of metabolic waste products. This effect could have been further enhanced by mineralization and metal precipitation on bacterial surfaces [56]. Adsorption of clay materials to bacteria may have occurred via extracellular polymeric substances (EPS) based on the formation of hydrogen bonds and electrostatic interactions [61].

Another mechanism of the antimicrobial activity of clays may be metal release. It has been hypothesized that the hydration of clays induces mineral oxidation, dissolution, and hydrolysis processes, resulting in an increase in acidity. Consequently, the lowered pH facilitates the release of soluble metal ions, including Al^3+^, Ca^2+^, and Fe^2+^, through the dissolution of clay mineral constituents [17,56,57]. Fe(II) ions present in clays are considered to be a key component responsible for the generation of reactive oxygen species (ROS) and antimicrobial activity. Studies showed that clays, especially those rich in iron, can generate ROS as a result of oxidation under the influence of atmospheric oxygen. The hydroxyl radical (•OH), in particular, with its high redox potential, is considered the most reactive ROS, capable of causing oxidative damage to biomolecules. Fe^2+^ ions, after penetrating the cell through a damaged membrane, also participate in reactions with endogenous H_2_O_2_ generated by cellular respiration, leading to the formation of intracellular hydroxyl radicals. Excessive production of these reactive molecules results in oxidative damage to cellular structures such as proteins and lipids [56,62,63]. Furthermore, intracellular oxidative stress can cause single-strand DNA breaks, inhibiting cell growth until DNA is repaired [43]. Additionally, metal ions can exert antimicrobial effects by impairing nutrient absorption or competing with essential enzyme cofactors [57,64]. All of these events ultimately lead to cell death. It should be noted that the MIC test is a typical screening test used to initially select the most effective compounds, whereas the direct contact test is recommended for testing various materials, including biomaterials, and it better reflects the conditions under which clays would be used as bioactive ingredients in dressing materials.

### 3.3. Cytotoxicity Assay

**Figure 6:**
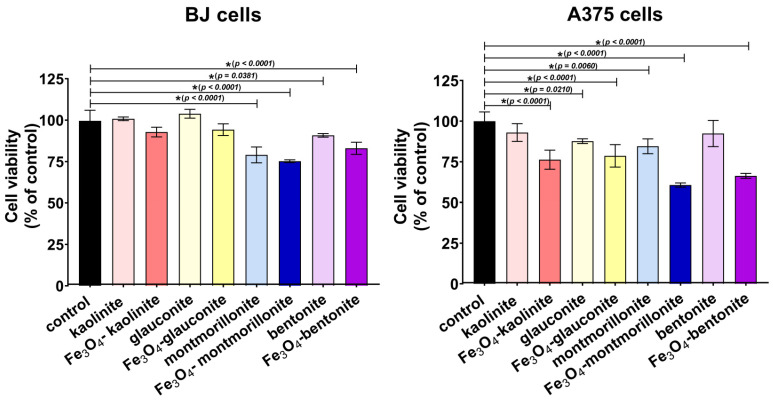
Cytotoxicity assessment against human normal fibroblasts (BJ cell line) and human melanoma cells (A375 cell line) using MTT assay after exposure to 24 h extracts of the tested materials; control—cells incubated in a polypropylene extract (negative control of cytotoxicity); * statistically significant differences compared to the control (*p* < 0.05, one-way ANOVA followed by Dunnett’s post hoc test).

Cytotoxicity assessment of the tested materials against human normal fibroblasts (BJ cell line) and human melanoma cells (A375 cell line) was performed using the MTT assay, following the ISO 10993-5 procedure (Figure 6). After 24 h of exposure to the material extracts, montmorillonite and Fe_3_O_4_–montmorillonite showed the greatest reduction in BJ cell viability (to 79% and 75%, respectively) compared to the other samples. According to ISO 10993-5, material extracts that reduce cell viability by less than 30% (i.e., viability retained above 70%) are considered as non-cytotoxic. Therefore, the tested clays and magnetite-modified clays can be classified as non-toxic in the human fibroblast in vitro model. Importantly, pure clays also showed selective cytotoxicity against melanoma cells, where the reduction in viability was generally greater than that observed for normal fibroblasts, except for kaolinite and bentonite, where cancer cell viability was above 90%. Modification of the clays with magnetite resulted in increased reduction in melanoma cell viability. The highest cytotoxicity in melanoma cells was observed for Fe_3_O_4_–montmorillonite and Fe_3_O_4_–bentonite (cell viability equal to 61% and 66%, respectively).

Tertiary chemoprevention is defined as the use of various agents to prevent cancer recurrence in patients after successful treatment of early disease [65]. Similarly, according to the definition provided by the National Cancer Institute of the USA [66], chemoprevention is the administration of certain drugs or other biologically active agents to reduce a risk of developing cancer or prevent its recurrence. Thus, it may be suggested that the developed magnetite–clay complexes show potential to be used as chemopreventive compounds in dressing materials that will exert cytotoxic effects towards remaining melanoma cells after tumor excision, reducing the risk of cancer recurrence. However, it should be noted that this paper presents only preliminary results and further research is needed to confirm this assumption. The promising potential of clays in anticancer therapy has also been noticed by other authors [67,68]. Nevertheless, it should be emphasized that in these papers the authors focused on clays as drug carriers and not on their own anticancer activity. To the best of our knowledge, there are no existing studies that examine the anticancer potential of clays per se, without their combination with therapeutic agents. Combining their reports with the results obtained by us, it can be assumed that the use of Fe_3_O_4_–montmorillonite or Fe_3_O_4_–bentonite as a carrier of an anticancer drug may have a high potential for success in anticancer therapy and should be taken into consideration.

The plausible mechanisms of action of biomaterials towards microbes and cancer cells are most often hard to fully describe. In many cases, the overall effect is the combination of many small details that occur during the interactions of the material with biological surfaces. Generally, it is known that the most common mechanisms of action are related to the adsorption of microorganisms or cells to materials and the interactions between them, the release of different metal ions through ionic exchange mechanisms and the disruption of metabolism, and catalytic reactions leading to the formation of reactive oxygen species (ROS) [69]. The antimicrobial properties and cytotoxicity against cancer cells of the magnetite–clay complexes probably do not come from a single process but rather from several that act together. For the antimicrobial activity, the most likely explanation is the formation of ROS at the magnetite surface. These ROS can damage membranes, proteins, and nucleic acids. The clays may add to this by providing a large surface that helps the particles attach to microbial cells, which makes the oxidative stress more effective. For cytotoxicity against cancer cells, ROS again seem central, but cancer cells are especially sensitive to oxidative stress, and the acidic conditions around tumors may also promote some release of iron from magnetite, which would further drive redox reactions. In addition, disturbance of the iron balance inside the cell and direct contact with clay platelets could help trigger cell death pathways. Taken together, the activity we see is best explained by a mix of ROS generation and surface-related interactions, though the relative weight of these effects may differ for microbes and cancer cells.

It should also be noted that modified clays may have the ability to promote the regeneration process of hard-to-heal wounds by eliminating factors responsible for their chronicity, such as microbial contamination, excessive exudates, and elevated level of matrix metalloproteinases (MMPs) [70]. Specifically, magnetite–clay complexes may have the ability to clean the wound bed of microorganisms via adsorbing pathogens and killing them and lowering the pH level of the wound bed, inhibiting elevated activity of MMPs. Moreover, the modified clays—due to their large specific surface area and high absorption abilities—could absorb excessive wound exudate and reveal hemostatic properties [70,71]. Clay minerals have also been proven to stimulate skin cell proliferation, contributing to tissue regeneration [72]. Therefore, it may be assumed that clay-based complexes may exhibit similar pro-healing properties. All of the above allows us to suggest that modified clays may potentially accelerate the healing process of chronic wounds.

## 4. Conclusions

The experiments clearly revealed that modification of natural clays with magnetite significantly boosted their antimicrobial properties without meaningful negative effects on the viability of human skin cells. This feature is highly advantageous in the context of potential applications of the investigated clays in patients for wound therapy. The lack of toxicity towards normal human fibroblasts (BJ cell line) significantly minimizes the potential risk of adverse effects of these clays on patients’ healthy tissues, while maintaining their antimicrobial and chemopreventive properties. Moreover, none of the reports known to us have examined both the antimicrobial activity of the tested clays and their toxicity towards normal human fibroblasts and cancer cells. Among the tested materials, Fe_3_O_4_–montmorillonite and Fe_3_O_4_–bentonite exhibited the strongest antimicrobial properties and cytotoxicity against cancer cells. The Fe_3_O_4_–montmorillonite complex appears to warrant particular attention. Its high activity may suggest a synergistic effect between the clay and the iron oxide phase, probably driven by ROS generation. These results are consistent with previous reports highlighting the antimicrobial role of Fe_3_O_4_ nanoparticles and some natural clays, but it is noteworthy that Fe_3_O_4_–clay complexes have a unique stand-alone anticancer and antimicrobial potential, regardless of drug loading. Therefore, due to the relative ease with which they can be modified with various chemical compounds, clays may serve as a useful basis for further research on drug-loaded systems. Overall, the antimicrobial activity and cytotoxicity against cancer cells of magnetite–clay complexes can be ascribed to a combination of mechanisms, with ROS generation at the magnetite surface playing the central role, supported by surface interactions with the clay matrix and, under certain conditions, limited iron release.

Taken together, these results provide a strong rationale for further development of magnetite-functionalized clays as advanced components of wound dressings, particularly in applications involving infected or post-oncological wounds. Their dual antimicrobial and anticancer action may help to reduce the risk of local recurrence after tumor excision while simultaneously preventing bacterial colonization. Taking into account the inherent magnetic responsiveness of the materials, we plan to continue this line of research and perform external stimulation of a magnetite–clay complex-loaded dressing material with Pulsed Electromagnetic Field (PEMF), which is known to support wound healing and tissue regeneration. Furthermore, our future research will involve activation of this complex-loaded dressing with Cold Atmospheric Plasma (CAP) to increase, via heterogeneous Fenton-like reactions, CAP-induced generation of ROS, enhancing the pro-healing, antimicrobial, and anticancer activity of the dressing dependent on the exposure time to CAP.

## Figures and Tables

**Table 1:** Antimicrobial activity of clays and clay–magnetite complexes presented as MIC [mg/mL] and MFC [mg/mL]. No minimum bactericidal concentration (MBC) was detected for bacteria in the tested concentration range.

Tested Clay Mineral	*E. coli*	*P. aeruginosa*	*S. aureus*	*C. albicans*	
	MIC	MIC	MIC	MIC	MFC
**kaolinite**	ND	ND	5	5	ND
**Fe_3_O_4_** **–** **kaolinite**	10	10	5	5	5
**glauconite**	ND	ND	2.5	20	ND
**Fe_3_O_4_** **–** **glauconite**	20	20	1.25	5	20
**montmorillonite**	ND	ND	10	20	20
**Fe_3_O_4_–montmorillonite**	0.156	0.156	0.156	1.25	1.25
**bentonite**	10	20	2.5	5	ND
**Fe_3_O_4_–bentonite**	5	5	0.313	2.5	ND

ND—not determined (no antimicrobial activity detected).

**Table 2:** Assessment of antibacterial activity of selected clays in direct contact tests according to the OECD standard.

Bacteria Strain	Mean of the Number of Viable Bacteria (CFU)				Antibacterial Activity	
	Control Material Ti t = 0	Control Material Ti t = 24 h	Tested Clay Mineral	Tested Materials	Log CFU Reduction	% CFU Reduction
***S. aureus***	1.31 × 10^4^	1.17 × 10^4^	montmorillonite	<1	4.07	99.99
			Fe_3_O_4_–montmorillonite	<1	4.07	99.99
			bentonite	3.76 × 10^4^	NA	NA
			Fe_3_O_4_–bentonite	1.41 × 10^4^	NA	NA
***E. coli***	4.14 × 10^4^	6.35 × 10^6^	montmorillonite	<1	6.8	99.99
			Fe_3_O_4_–montmorillonite	<1	6.8	99.99
			bentonite	5.06 × 10^6^	0.1	20.31
			Fe_3_O_4_–bentonite	2.42 × 10^5^	1.42	96.19

NA—no activity (the number of bacteria was equal to or greater than that on the control material).

## Data Availability

The datasets generated during and/or analyzed during the current study are available in the Mendeley Data repository, https://doi.org/10.17632/rj6rsnvt2j.1.

## Abbreviations

The following abbreviations are used in this manuscript:

ATCC	American Type Culture Collection
CAP	Cold Atmospheric Plasma
DNA	Deoxriboynucleic acid
EUCAST	European Committee on Antimicrobial Susceptibility Testing
MBC	Minimum bactericidal concentration
MFC	Minimum fungicidal concentration
MGO	Methyloglyoxal
MIC	Minimum inhibitory concentration
MMPs	matrix metalloproteinases
MTT	3-(4,5-dimethylthiazol-2-yl)-2,5-diphenyltetrazolium bromide
ND	Not determined
PEMF	Pulsed Electromagnetic Fields
PET	Polyethylene terephthalate
PVP	Polivinyl pyrrolidone
ROS	Reactive oxygen species
SD	Standard deviation
SEM	Scanning electron microscopy
TEM-EDS	Transmission electron microscopy with energy-dispersive X-ray spectroscopy
XRD	X-ray diffraction
